# Odd Response-Induced Phase Separation of Active Spinners

**DOI:** 10.34133/research.0356

**Published:** 2024-05-03

**Authors:** Yu Ding, Boyi Wang, Qing Yang, Zhiyuan Zhao, Shigeyuki Komura, Ryohei Seto, Mingcheng Yang, Fangfu Ye

**Affiliations:** ^1^ Beijing National Laboratory for Condensed Matter Physics and Laboratory of Soft Matter Physics, Institute of Physics, Chinese Academy of Sciences, Beijing 100190, China.; ^2^ School of Physical Sciences, University of Chinese Academy of Sciences, Beijing 100049, China.; ^3^ Wenzhou Institute, University of Chinese Academy of Sciences, Wenzhou, Zhejiang 325001, China.; ^4^ Oujiang Laboratory (Zhejiang Lab for Regenerative Medicine, Vision and Brain Health), Wenzhou, Zhejiang 325000, China.; ^5^ Department of Chemistry, Graduate School of Science, Tokyo Metropolitan University, Hachioji, Tokyo 192-0397, Japan.; ^6^ Graduate School of Information Science, University of Hyogo, Kobe, Hyogo 650-0047, Japan.; ^7^ Songshan Lake Materials Laboratory, Dongguan, Guangdong 523808, China.

## Abstract

Due to the breaking of time-reversal and parity symmetries and the presence of non-conservative microscopic interactions, active spinner fluids and solids respectively exhibit nondissipative odd viscosity and nonstorage odd elasticity, engendering phenomena unattainable in traditional passive or active systems. Here, we study the effects of odd viscosity and elasticity on phase behaviors of active spinner systems. We find the spinner fluid under a simple shear experiences an anisotropic gas–liquid phase separation driven by the odd-viscosity stress. This phase separation exhibits equilibrium-like behavior, with both binodal-like and spinodal curves and critical point. However, the formed dense liquid phase is unstable, since the odd elasticity instantly takes over the odd viscosity to condense the liquid into a solid-like phase. The unusual phase behavior essentially arises from the competition between thermal fluctuations and the odd response-induced effective attraction. Our results demonstrate that the cooperation of odd viscosity and elasticity can lead to exotic phase behavior, revealing their fundamental roles in phase transition.

## Introduction

Classical fluid and elastic mechanics are based on certain symmetry and conservation laws, ensuring that linear response coefficients therein are symmetric. When the constraints are broken, antisymmetric response coefficients may emerge, such as odd viscosity and elasticity [1]. Odd viscosity represents the antisymmetric part of viscosity tensor, $\eta_{\text{ijkl}}^{o}=-\eta_{\text{klij}}^{o}$, which arises from the breaking of time-reversal symmetry [2–5]. The odd viscosity, also known as Hall viscosity, has been studied across many scales, including quantum Hall fluids [6], superfluids [7], polyatomic gases [8], magnetized plasmas [9], and chiral active fluids [10–13]. On the other hand, odd elasticity refers to the antisymmetric elastic modulus, $K_{\text{ijkl}}^{o}=-K_{\text{klij}}^{o}$, which results from non-conservative microscopic interactions [14]. Such odd elastic solids can be constructed from active hinges and metamaterials [15,16], spinners [17,18], or muscle fibers [19].

Unlike traditional dissipative viscosity and storage elasticity, the odd viscosity is dissipationless and the odd elasticity is nonstored [1]. The odd response coefficients have far-reaching consequences for the physical properties of materials and often engender unexpected phenomena. For instance, the odd viscosity remarkably affects the velocity field of the fluid [20,21] and the forces/torques on embedded objects [12,22–25], induces the transverse mass transport [26,27] and topological sound waves at the fluid boundary [28], and markedly changes the Kelvin–Helmholtz and Saffman–Taylor instabilities of the fluid flows [29,30]. On the other hand, the odd elasticity fundamentally alters the elastostatics and elastodynamics of the solids [14], even generates non-zero work over a quasistatic closed cycle deformation [14], induces topological edge modes [31], and causes self-kneading whorl structure in chiral crystals [18,32]. Despite the great progresses, the existing works focus on the existence of the odd viscosity and elasticity and their effects on the mechanics, transport, and dynamics of the materials. In particular, the odd viscosity and odd elasticity are investigated separately. However, the effect of the odd response coefficients on phase behavior is rarely explored, especially when both the odd viscosity and elasticity are essential.

Here, we perform particle-based simulations to study the phase behavior of a 2-dimensional (2D) fluid of repulsive active spinners in an external shear, concentrating on the role of the odd viscosity and elasticity. The reason for considering the spinner system is that it exhibits the odd viscosity in the fluid phase [5,10,12,13] and the odd elasticity in the solid phase [17,18], hence serving as an ideal model. We find that the odd viscous stress triggers an equilibrium-like gas–liquid phase separation in the direction perpendicular to the shear, while remaining uniform along the shear. Further, once the dense liquid phase occurs, the odd elastic stress plays a role to condense the liquid into a solid-like phase, which is stable only when its width is within an activity-dependent range. All the findings can be quantitatively explained by continuum theories.

## Results

**Fig. 1.**
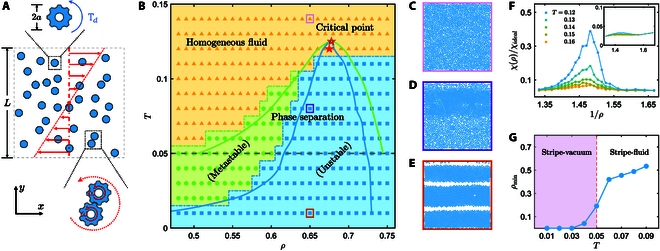
(A) Schematic of interacting active spinners under simple shear (red arrows). (B) Phase diagram: colored solid symbols correspond to the simulation results, and the blue and green curves are, respectively, the spinodal and binodal-like curves obtained theoretically (see Fig. 2B). The solid and open stars refer to the critical point determined separately from the simulation measurement of compressibility and theory (Fig. 2B). (C to E) Representative snapshots of particle configurations at *T* = 0.14 (C), *T* = 0.08 (D), and *T* = 0.01 (E), with *ρ* = 0.65. (F) Relative compressibility of the homogeneous spinner fluid as a function of 1/*ρ* for various *T*. The main diagram and inset display *χ*/*χ*_ideal_ in the *y* and *x* directions, respectively. (G) The minimum packing fraction of the coexisting dilute phase vs. temperature.

The system consists of an ensemble of active spinners of radius *a* in a square box of size *L* = 100*a*, subjected to a simple shear $\dot{\gamma }$ (Fig. 1A). The translational and rotational degrees of freedom of the spinners evolve according to Langevin equations,

$$m{\dot{v}}_{i}=F_{p}+F_{\text{ex}}+\zeta -\gamma_{t}v_{i},$$ (1)

Iω˙i=Td+ξ−γrωi, (2)

with *m*, *I* = *ma*^2^/2, *γ_t_*, and *γ_r_* = 4*a*^2^*γ_t_*/3 separately being the mass, moment of inertia, and translational and rotational friction coefficients. Here, T_d_ is the driving torque, and $F_{\text{ex}}=\dot{\gamma }\gamma_{t}\left(y-L/2\right)\hat{x}$ denotes the external force field, combined with the Lees–Edwards boundary, to generate a uniform shear in the dissipative environment. The terms ***ζ*** and *ξ* are zero-mean Gaussian white noises of variance 〈***ζ***(*t*)***ζ***(*t*^′^)〉 = 2*k*_B_*Tγ_t_δ*(*t* − *t*^′^)**1** and 〈*ξ*(*t*)*ξ*(*t*^′^)〉 = 2*k*_B_*Tγ_r_δ*(*t* − *t*^′^), with the temperature *T*. Different spinners interact through the Weeks–Chandler–Andersen-type repulsion **F**_p_ with the characteristic energy ϵ, and at the same time, they couple tangentially via interparticle friction that is achieved by instantaneously updating **v***_i_* and *ω_i_* according to the relative velocity at the impact point of 2 rough disks [33]. The units of length, energy, and mass are separately given by *a*, ϵ, and *m*, producing the unit of time $t_{0}=\sqrt{{\mathrm{ma}}^{2}/\epsilon }$ and the unit of temperature ϵ/*k*_B_. In simulations, the frictional coefficients ($\gamma_{t}=100\sqrt{m\epsilon /a^{2}}$) are sufficiently high to make inertia negligible. More details are given in the Supplementary Materials.

### Phase behavior of the sheared spinner fluid

To directly and thoroughly compare with equilibrium phase transitions, which are usually investigated in the *T* − *ρ* plane without shear and active torque, we fix $\dot{\gamma }$ and T_d_ and change *ρ* and *T*. We take $\dot{\gamma }t_{0}=10^{-3}$ and T_d_/ϵ = 40 (counterclockwise), which not only lead to remarkable odd response but ensure the system in the linear response regime (see the Supplementary Materials).

Figure 1B displays the *T* − *ρ* phase diagram of the sheared spinner fluid. The fluid is either homogeneous or phase-separated, depending on *T* and *ρ*. The representative snapshots of different states are presented in Fig. 1C to E, manifesting that the phase separation only happens in the direction perpendicular to the shear. The phase-separated region can be divided into 2 different domains, in which the homogeneous fluid is, respectively, unstable and metastable (see the Supplementary Materials). In the unstable regime, the homogeneous fluid spontaneously phase-separates, while the metastable fluid keeps its initialized (homogeneous or phase-separated) state in the simulation duration. Note that the transition density of the spinner fluid may be much less than that of the equilibrium 2D repulsive disks [34], which is about *ρ* = 0.74.

Following the concept of equilibrium gas–liquid phase transition, the borderline between the homogeneous and phase-separated regions corresponds to the binodal-like curve, and the metastable and unstable domains are separated by the spinodal curve. Moreover, in equilibrium, the coincidence of the spinodal and binodal curves implies a critical point, at which a system displays a singular behavior [35]. To see if there exists such a critical point in the out-of-equilibrium spinner system, we quantify the relative compressibility of the spinner fluid by measuring the particle number fluctuations in a subregion of the system [36],*χ*/*χ*_ideal_ = (〈*N*^2^〉 − 〈*N*〉^2^)/  〈N〉 (see the Supplementary Materials). As plotted in Fig. 1F, the compressibility perpendicular to the shear develops a peak when approaching the solid star in Fig. 1B, reminiscent of the equilibrium critical point. In contrast, *χ*/*χ*_ideal_ is almost unchanged in the direction of the shear, reflecting the anisotropy of phase separation.

Besides its anisotropy, the structure of the final coexisting phases in these odd-response active spinners differs from that of the equilibrium gas–liquid system. Here, the dense phase is closer to a solid stripe with a hexagonal lattice structure than to a liquid, especially in a low-temperature regime. A moderately wide stripe stably moves as a whole entrained by the shear flow, while a sufficiently wide stripe continuously fragments and reassembles, similar to the self-kneading crystal [18]. The coexisting dilute phase may even be “vacuum”. The solid-like stripe and the vacuum state possibly coexist only for the temperature *T* ≲ 0.05, as Fig. 1G indicates that the minimum density of the coexisting dilute phase experiences a remarkable rise around *T* = 0.05. We emphasize that the phase separation of active spinners is fundamentally different from the shear-induced aggregation or band of passive colloids and polymers. The latter usually originates from nonlinear effects of traditional (even) viscoelasticity [37] and takes place for both positive and negative shear. In contrast, the present case only occurs for negative shear with respect to the spinning direction and will be shown to arise from the linear odd viscosity and odd elasticity.

Despite the unusual phase behavior, the existence of the phase-separated region, metastable domain, and critical point suggests that the phase separation of the sheared spinners may share the same mechanism as equilibrium gas–liquid transition. Following the standard route to study the equilibrium gas–liquid transition by the equation of state of a uniform fluid, we will investigate the phase separation of the sheared spinner fluid by analyzing its normal stress.

### Odd viscosity-driven anisotropic phase separation

According to the 2D continuum hydrodynamic theory of the spinner fluid [3,10–12], the stress $\sigma_{\mathrm{ij}}^{f}$ and the strain rate *∂_i_v_j_* fulfill the constitutive relation,

σijf=−pδij+σijV=−pδij+η∂ivj+∂jvi−δij∂kvk+ζδij∂kvk+ηRϵij2ω−Ω+ηo∂iϵjkvk+ϵik∂kvj, (3)

with *p* being the pressure, ϵ*_ij_* being the Levi–Civita symbol, *ω*(**r**, *t*) being the angular velocity field, and $\Omega =\hat{z}\cdot \left(\nabla \times v\right)$ being the vorticity of flow field. Here, the second and third terms are ordinary viscous stresses with the shear viscosity *η* and bulk viscosity *ζ*; the fourth term, with *η_R_* being he rotational viscosity, refers to the antisymmetric stress that couples the spin and flow; the last one is the nondissipative stress from the odd viscosity *η_o_*.

Under an external shear *∂_y_v_x_* > 0 (Fig. 1A), Eq. 3 indicates that the odd viscosity contributes additional anisotropic normal stresses, which are separately $\sigma_{\mathrm{xx}}^{V,o}=\eta_{o}\partial_{y}v_{x}$ and $\sigma_{\mathrm{yy}}^{V,o}=\;-\eta_{o}\partial_{y}v_{x}$ along the *x* and *y* directions. As *η_o_* is negative for T_d_ > 0 [12], $\sigma_{\mathrm{xx}}^{V,o}$ and $\sigma_{\mathrm{yy}}^{V,o}$ amount to effective repulsion and attraction, respectively. It is the competition between the pressure and $\sigma_{\mathrm{yy}}^{V,o}$ that drives the spinners to phase-separate in the *y* direction. For convenience, we regard $-\;\sigma_{\mathrm{yy}}^{f}$ as the effective pressure,

$$p_{\text{eff}}^{f}=-\;\sigma_{\mathrm{yy}}^{f}=p+\eta_{o}\partial_{y}v_{x}.$$ (4)

**Fig. 2.**
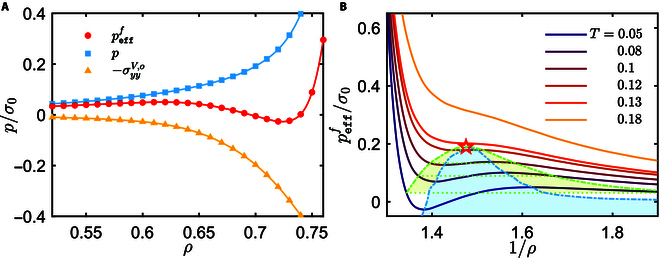
(A) Normal stress (in units of $\sigma_{0}=m/t_{0}^{2}$) vs. *ρ*, with *T* = 0.05. (B) Effective pressure of the uniform spinner fluid (clockwise) exhibits a van der Waals-like equation of state. Here, the horizontal dashed lines denote the Maxwell constructions; the obtained binodal curve (green dashed–dotted line), spinodal curve (blue dashed–dotted line), and critical point (open star) are also plotted in Fig. 1B.

Here, *p* and *η_o_* can be determined for various *ρ* from independent simulations with reverse-rotating spinners (clockwise), in which the spinner fluid is spatially homogeneous (see the Supplementary Materials). As shown in Fig. 2A, the magnitude of *p* and $\sigma_{\mathrm{yy}}^{V,o}$ increases with *ρ*, resulting in a non-monotonic dependence of $p_{\text{eff}}^{f}$ on *ρ*. Clearly, the uniform fluid is mechanically unstable in the domain with the condition

$$\frac{\partial p_{\text{eff}}^{f}}{\partial \rho }<0,$$ (5)

where a minor density deviation from the mean *ρ* triggers the spinodal decomposition. The *ρ* dependence of $p_{\text{eff}}^{f}$ for varying temperatures is plotted in Fig. 2B, resembling the van der Waals equation of state. The relation $\partial p_{\text{eff}}^{f}/\partial \rho =0$ determines the spinodal curves. At zero temperature, our previous numerical work shows that athermal rotors also form stripes under shear [13], which, however, is explained in terms of deterministic interparticle microscopic collision dynamics. Considering the odd viscosity and the non-monotonic effective pressure, it is now clear that the sheared athermal rotor system is mechanically unstable. When either torque or shear is reversed, the spinner fluid will be homogeneous, since $\sigma_{\mathrm{yy}}^{V,o}<0$ (effectively repulsive). Although $\sigma_{\mathrm{xx}}^{V,o}>0$ (attractive) in this situation, the shear flow impedes the *x*-direction phase separation of the spinner fluid.

To understand the existence of the metastable spinner fluid, we notice that chiral active fluids often exhibit equilibrium-like characteristics in their translational degrees of freedom. For example, spinner fluids are recently shown to display a Maxwell distribution of translational velocities, a Boltzmann distribution of particle concentration within an external potential, and to satisfy a fluctuation–dissipation relation for their viscous response [5]. In addition, a chiral active binary mixture can undergo an equilibrium-like transition similar to a thermal binary fluid [38]. Furthermore, Han et al. [5] demonstrated that the equilibrium-like behaviors of the chiral active fluids arise from the fact that the fluctuating (translational) and activated (rotational) degrees of freedom of the system are statistically decoupled (negligible mutual information), such that the fluctuating degrees of freedom behave equilibrium-like. These observations suggest that it is possible to use equilibrium thermodynamics to study the phase behavior of the nonequilibrium spinner fluid. Based on the above arguments, we utilize the Maxwell’s equal-area rule to construct the border for the phase separation (binodals) and to further identify the critical point (open star), as plotted in Fig. 2B.

For comparison, the predicted spinodal and binodal curves and critical point are drawn in Fig. 1B. Remarkably, the critical point and the low-density branches of the spinodal and binodal curves well match the phase diagram obtained from the direct simulations. Thus, the phase behavior of the spinners may be reasonably described in the framework of equilibrium gas–liquid transition, when the odd viscous stress is considered. However, it should be noted that the final coexisting states in the direct simulations are solid-like phase and fluid instead of 2 fluids with different densities, and that the dense spinner liquids are unstable. As a result, the high-density branches of spinodal and binodal curves are lacking (Fig. 1B), unlike the above theoretical prediction (Fig. 2B). The differences imply that the fluid theory is invalid in the dense regime, in which the elasticity could take effect.

### Odd elasticity-reshaped coexisting phases

To understand the abnormal coexisting states of the phase-separated spinners, we account for the elastic effect, which becomes considerable at high densities [14,18,39]. Since the 2D solid composed of active spinners is an innate odd elastic material [17,18], its stress is of the form,

σijs=−pδij+2ηRωϵij+σijE=−pδij+2ηRωϵij+μ∂iuj+∂jui−δij∂kuk+λδij∂kuk+Ko∂kϵikuj+∂iϵjkuk, (6)

with the local displacement field **u**(**r**, *t*) that is related to the local velocity field **v**(**r**, *t*) in Eq. 3 by **v** = *∂_t_***u**. Here, the spin-induced asymmetric stress 2*η_R_ω*ϵ*_ij_* still exists [14,18]. In the elastic stress part $\sigma_{\mathrm{ij}}^{E}$, *λ* and *μ* are, respectively, the bulk and shear elastic moduli, and *K_o_* refers to the odd elastic modulus. To see the odd elastic effect, we consider a periodic hexagonal crystal of spinners under a shear strain *∂_y_u_x_*. In this case, the odd elasticity produces anisotropic normal stresses that are $\sigma_{\mathrm{xx}}^{E,o}=K_{o}\partial_{y}u_{x}$ and $\sigma_{\mathrm{yy}}^{E,o}=-\;K_{o}\partial_{y}u_{x}$. Similarly, we define an effective pressure in the *y* direction for the spinner solid,

$$p_{\text{eff}}^{s}=p-\sigma_{\mathrm{yy}}^{E,o}=p+K_{o}\partial_{y}u_{x}.$$ (7)

**Fig. 3.**
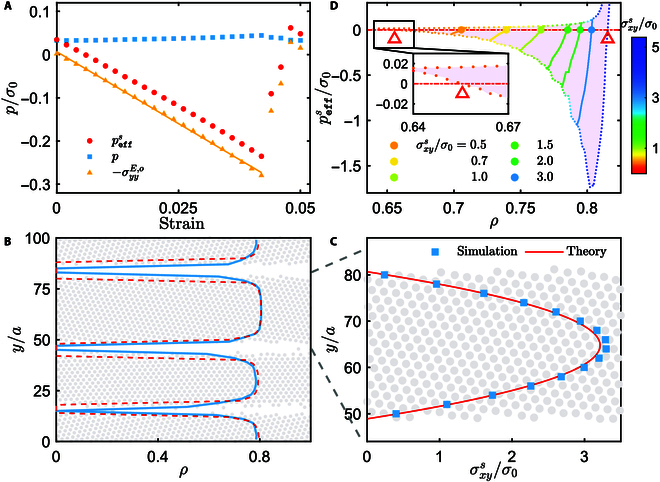
(A) Normal stress–strain relation of the spinner crystal at *T* = 0.01 and *ρ* = 0.75, yielding *K_o_* =  − 6.65 by fitting. (B) Packing fraction and (C) tangential stress distributions of the emerging stripes at *T* = 0.01 and *ρ* = 0.65, where the blue data refer to the simulation and the red lines refer to the theoretical predictions from Eqs. 8 and 9. (D) Effective pressure of the spinner crystal vs. *ρ* for various $\sigma_{\mathrm{xy}}^{s}$ (colored solid lines), where $p_{\text{eff}}^{s}\left(\rho ;\sigma_{\mathrm{xy}}^{s}\right)=0$ determines the *ρ* distribution in the stripe [red dashed line in (B)]. Here, the upper and lower dotted lines, respectively, correspond to the zero and maximum (elastic limit) shear deformations, and the open triangles mark the region in which $p_{\text{eff}}^{s}\left(\rho ;\sigma_{\mathrm{xy}}^{s}\right)=0$ is solvable.

Figure 3A, obtained from simulation, shows that before plastic deformation, the odd elastic term linearly increases with the strain, having a negative slope *K_o_*, while *p* remains constant. Similar to the role of *η_o_* in the spinner fluid, the odd elastic stress $\sigma_{\mathrm{yy}}^{E,o}>0$ produces effective attractions along the *y* direction in the spinner solid.

Based on the above observation, once the (otherwise stable) coexisting dense liquid emerges from the sheared spinners, $\sigma_{\mathrm{yy}}^{E,o}$ exerts an extra attraction that breaks the balance between $\sigma_{\mathrm{yy}}^{V,o}$ and pressure, condensing the dense liquid into the solid stripe and markedly reshaping the coexisting phases. Here, the formation of the odd elastic solid results from the odd elasticity itself, in contrast to the cases of magnetic colloidal rotors [18] and starfish embryos [17], where additional magnetic or hydrodynamic attraction is needed.

We now use the elastic theory to quantitatively study the properties of the final coexisting states. For simplicity, we first consider the solid-vacuum coexisting state, where $p_{\text{eff}}^{s}$ vanishes everywhere. In the shear flow, the solid stripe moves as a whole at a constant velocity *v*_0_ along the *x* axis and suffers from a nonuniform shear strain. *v*_0_ is determined by balancing the external driving and environmental friction on the stripe, $\frac{1}{V_{p}}\int_{y_{0}-w_{s}}^{y_{0}+w_{s}}\;\left[f\left(y^{\prime }\right)-\gamma_{t}v_{0}\right]\rho \left(y^{\prime }\right)dy^{\prime }=0$, with *y*_0_ and *w_s_* separately being the center and half-width of the stripe, *V_p_* = *πa*^2^, and $f\left(y\right)=\dot{\gamma }\gamma_{t}\left(y-L/2\right)$ being the external force field. The linear *y* dependence of *f*(*y*) thus means that *v*_0_ is just the local velocity of the shear flow at the stripe center, namely, *γ_t_v*_0_ = *f*(*y*_0_). Further, the steady-state force balance on an element of the stripe in the *x*-direction is $0=\partial_{y}\sigma_{\mathrm{xy}}^{s}\left(y\right)+\left[f\left(y^{\prime }\right)-f\left(y_{0}\right)\right]\rho \left(y^{\prime }\right)/V_{p}$. By integration with $\sigma_{\mathrm{xy}}^{s}\left(y_{0}+w_{s}\right)=0$ (at the stripe upper edge), the local tangential stress reads

$$\sigma_{\mathrm{xy}}^{s}\left(y\right)=\frac{1}{V_{p}}\int_{y}^{y_{0}+w_{s}}\;\left[f\left(y^{\prime }\right)-f\left(y_{0}\right)\right]\rho \left(y^{\prime }\right){\mathrm{dy}}^{\prime },$$ (8)

indicating that $\sigma_{\mathrm{xy}}^{s}\left(y\right)$ monotonically increases from zero at the stripe edge to a maximum at the stripe center. Moreover, for any given *y* inside the stripe, $\sigma_{\mathrm{xy}}^{s}\left(y\right)$ increases with *w_s_*, since ∣*f*(*y*) − *f*(*y*_0_)∣ can take a larger value for a wider stripe. Approximating *ρ*(*y*) as a constant value 0.8 (close to the mean *ρ* of the stripe [Fig. 3B]), Eq. 8 reproduces the tangential stress obtained from the direct simulation (Fig. 3C).

Actually, the local density of the stripe is position-dependent. By spontaneous relaxation of *ρ*(*y*), the effective pressure (Eq. 7) reaches zero throughout the stripe,

$$p_{\text{eff}}^{s}\left(y\right)=p\left(y\right)+K_{o}\frac{\sigma_{\mathrm{xy}}^{s}\left(y\right)-2\eta_{R}\omega }{\mu }=0,$$ (9)

where $\sigma_{\mathrm{xy}}^{s}\left(y\right)=2\eta_{R}\omega +\mu \partial_{y}u_{x}$ is used. The combination of Eqs. 8 and 9 determines the density distribution of the stripe. It is highly nontrivial to solve the equations, since all the elastic moduli and pressure depend on *ρ*. Thus, we perform independent simulations to tentatively seek the solution, in which we shear a uniform periodic spinner crystal and tune its density until $p_{\text{eff}}^{s}\left(\rho ;\sigma_{\mathrm{xy}}^{s}\right)=0$ for various $\sigma_{\mathrm{xy}}^{s}$, as shown in Fig. 3D. The obtained *ρ* is a function of $\sigma_{\mathrm{xy}}^{s}$, which, together with the *y*–$\sigma_{\mathrm{xy}}^{s}\left(y\right)$ relation in Fig. 3C, gives the density distribution of the stripe, agreeing with the direct simulation measurement in the phase-separated system (Fig. 3B).

**Fig. 4.**
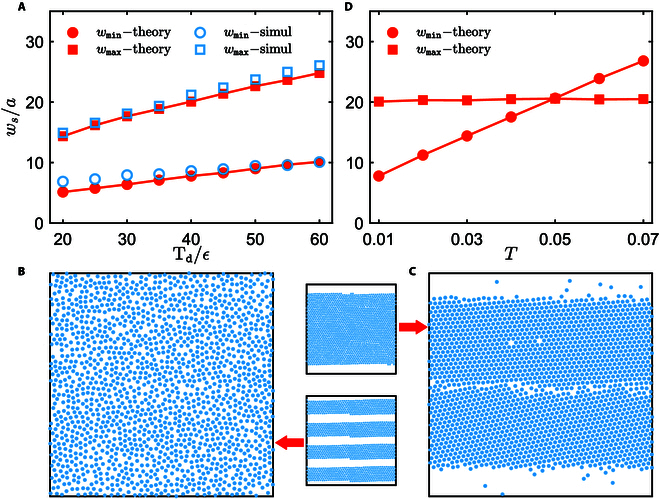
(A) Comparison of the maximum and minimum widths of the stable solid stripe obtained from the simulation and theory, for various T_d_ at *T* = 0.01. (B) and (C) represent simulation snapshots from initial solid stripe configurations (insets) of different widths, at *T* = 0.02 and T_d_/ϵ = 40. (D) The predicted maximum and minimum widths of the stable solid stripe as a function of temperature, with T_d_/ϵ = 40.

Nevertheless, the solution of Eq. 9 does not exist for very small or large $\sigma_{\mathrm{xy}}^{s}$ within the elastic limit of the spinner crystal, as displayed in Fig. 3D. Because $\sigma_{\mathrm{xy}}^{s}\left(y\right)$ increases monotonically with the stripe width (Eq. 8), the solvability condition with respect to $\sigma_{\mathrm{xy}}^{s}$ gives the width range of the solid stripe that can stably coexist with the vacuum (see the Supplementary Materials). The predicted width range of the stable stripe nicely agrees with the direct simulation measurement (Fig. 4A). Intuitively, for very narrow stripes (small $\sigma_{\mathrm{xy}}^{s}$), the odd elasticity effect is too weak to stabilize the stripe (Fig. 4B), while for very wide stripes, $\sigma_{\mathrm{xy}}^{s}$ at the stripe center is strong enough to destroy the solid structure (Fig. 4C). Furthermore, the current discussion shows that the minimum width is wider than the maximum one when *T* > 0.05 (Fig. 4D), meaning the stripe-vacuum coexisting state is unstable. In this case, the dilute coexisting phase is a fluid, consistent with the direct simulation (Fig. 1B and G).

## Conclusion

Using simulations and continuum theories, we study the phase behavior of sheared active spinner systems. We demonstrate that the odd viscosity initially drives an anisotropic equilibrium-like gas–liquid phase separation and the odd elasticity dramatically reshapes the final coexisting phases, thus revealing the important role of the odd responses in phase transition of chiral active matter. Interestingly, the onset of the phase separation of the active spinner fluid can be reasonably described in the framework of equilibrium phase transition, probably arising from the statistical decoupling between the fluctuating and activated degrees of freedom. Our findings could be verified with synthetic active rotors [10,11,40–43] or biological spinners [17,44,45]. Self-organization is prevalent in both linear [46–49] and chiral [17,50,51] active matter, playing a vital role in their functionality. Our work elucidates the mechanism underlying the self-organization of chiral active matter under shear, and thus provides insight for exploiting their potential applications in biomedicine and materials science.

## Data Availability

All data needed in the paper are present in the paper and in Supplementary Materials. Additional data related to this paper may be requested from the authors.

## References

[1] Fruchart M, Scheibner C, Vitelli V. Odd viscosity and odd elasticity. Annu Rev Condens Matter Phys. 2023;14:471–510.

[2] Avron JE. Odd viscosity. J Stat Phys. 1998;92:543–557.

[3] Banerjee D, Souslov A, Abanov AG, Vitelli V. Odd viscosity in chiral active fluids. Nat Commun. 2017;8:1573. 29146894 10.1038/s41467-017-01378-7 PMC5691086

[4] Markovich T, Lubensky TC. Odd viscosity in active matter: Microscopic origin and 3d effects. Phys Rev Lett. 2021;127: Article 048001. 34355935 10.1103/PhysRevLett.127.048001

[5] Han M, Fruchart M, Scheibner C, Vaikuntanathan S, De Pablo JJ, Vitelli V. Fluctuating hydrodynamics of chiral active fluids. Nat Phys. 2021;17:1260–1269.

[6] Avron J, Seiler R, Zograf PG. Viscosity of quantum hall fluids. Phys Rev Lett. 1995;75:697. 10060091 10.1103/PhysRevLett.75.697

[7] Read N. Non-abelian adiabatic statistics and hall viscosity in quantum hall states and p_x_+ip_y_ paired superfluids. Phys Rev B. 2009;79(4): Article 045308.

[8] Knaap H, Beenakker J. Heat conductivity and viscosity of a gas of non-spherical molecules in a magnetic field. Physica. 1967;33(3):643–670.

[9] Braginskii SI. Transport phenomena in a completely ionized two-temperature plasma. JETP. 1958;6:358.

[10] Soni V, Bililign ES, Magkiriadou S, Sacanna S, Bartolo D, Shelley MJ, Irvine WTM. The odd free surface flows of a colloidal chiral fluid. Nat Phys. 2019;15:1188–1194.

[11] Tsai JC, Ye F, Rodriguez J, Gollub JP, Lubensky T. A chiral granular gas. Phys Rev Lett. 2005;94(21):214301. 16090323 10.1103/PhysRevLett.94.214301

[12] Yang Q, Zhu H, Liu P, Liu R, Shi Q, Chen K, Zheng N, Ye F, Yang M. Topologically protected transport of cargo in a chiral active fluid aided by odd-viscosity-enhanced depletion interactions. Phys Rev Lett. 2021;126(19):198001. 34047594 10.1103/PhysRevLett.126.198001

[13] Zhao Z, Wang B, Komura S, Yang M, Ye F, Seto R. Emergent stripes of active rotors in shear flows. Phys Rev Research. 2021;3: Article 043229.

[14] Scheibner C, Souslov A, Banerjee D, Surówka P, Irvine WTM, Vitelli V. Odd elasticity. Nat Phys. 2020;16(4):475–480.

[15] Brandenbourger M, Scheibner C, Veenstra J, Vitelli V, Coulais C. Limit cycles turn active matter into robots. arXiv. 2021. https://arxiv.org/abs/2108.08837

[16] Chen Y, Li X, Scheibner C, Vitelli V, Huang G. Realization of active metamaterials with odd micropolar elasticity. Nat Commun. 2021;12:5935. 34642324 10.1038/s41467-021-26034-z PMC8511045

[17] Tan TH, Mietke A, Li J, Chen Y, Higinbotham H, Foster PJ, Gokhale S, Dunkel J, Fakhri N. Odd dynamics of living chiral crystals. Nature. 2022;607:287–293. 35831595 10.1038/s41586-022-04889-6

[18] Bililign ES, Balboa Usabiaga F, Ganan YA, Poncet A, Soni V, Magkiriadou S, Shelley MJ, Bartolo D, Irvine WTM. Motile dislocations knead odd crystals into whorls. Nat Phys. 2022;18:212–218.

[19] Shankar S, Mahadevan L. Active muscular hydraulics. bioRxiv. 2022. https://www.biorxiv.org/content/10.1101/2022.02.20.481216v1.full

[20] Khain T, Scheibner C, Fruchart M, Vitelli V. Stokes flows in three-dimensional fluids with odd and parity-violating viscosities. J Fluid Mech. 2022;934:A23.

[21] Holder T, Queiroz R, Stern A. Unified description of the classical hall viscosity. Phys Rev Lett. 2019;123:106801. 31573291 10.1103/PhysRevLett.123.106801

[22] Ganeshan S, Abanov AG. Odd viscosity in two-dimensional incompressible fluids. Phys Rev Fluids. 2017;2: Article 094101.

[23] Hosaka Y, Komura S, Andelman D. Hydrodynamic lift of a two-dimensional liquid domain with odd viscosity. Phys Rev E. 2021;104: Article 064613. 35030884 10.1103/PhysRevE.104.064613

[24] Hosaka Y, Komura S, Andelman D. Nonreciprocal response of a two-dimensional fluid with odd viscosity. Phys Rev E. 2021;103(4–1): Article 042610. 34005895 10.1103/PhysRevE.103.042610

[25] Yang Q, Liang H, Liu R, Chen K, Ye F, Yang M. Edge transport and self-assembly of passive objects in a chiral active fluid. Chin Phys Lett. 2021;38:128701.

[26] Berdyugin AI, Xu S, Pellegrino F, Krishna Kumar R, Principi A, Torre I, Ben Shalom M, Taniguchi T, Watanabe K, Grigorieva IV, et al. Measuring hall viscosity of graphene’s electron fluid. Science. 2019;364(6436):162–165. 30819929 10.1126/science.aau0685

[27] Lou X, Yang Q, Ding Y, Liu P, Chen K, Zhou X, Ye F, Podgornik R, Yang M. Odd viscosity-induced hall-like transport of an active chiral fluid. Proc Natl Acad Sci U S A. 2022;119(42): Article e2201279119. 36215475 10.1073/pnas.2201279119 PMC9586265

[28] Souslov A, Dasbiswas K, Fruchart M, Vaikuntanathan S, Vitelli V. Topological waves in fluids with odd viscosity. Phys Rev Lett. 2019;122(12):128001. 30978035 10.1103/PhysRevLett.122.128001

[29] Faganello M, Califano F. Magnetized kelvin–Helmholtz instability: Theory and simulations in the Earth’s magnetosphere context. J Plasma Phys. 2017;83(6):535830601.

[30] Reynolds D, Monteiro GM, Ganeshan S. Hele-Shaw flow for parity odd three-dimensional fluids. Phys Rev Fluids. 2022;7:114201.

[31] Scheibner C, Irvine WT, Vitelli V. Non-Hermitian band topology and skin modes in active elastic media. Phys Rev Lett. 2020;125:118001. 32976010 10.1103/PhysRevLett.125.118001

[32] Braverman L, Scheibner C, VanSaders B, Vitelli V. Topological defects in solids with odd elasticity. Phys Rev Lett. 2021;127:268001. 35029487 10.1103/PhysRevLett.127.268001

[33] Allen M, Tildesley D. *Computer simulation of liquids*. USA: Oxford University Press; 1989.

[34] Bernard EP, Krauth W. Two-step melting in two dimensions: First-order liquid-hexatic transition. Phys Rev Lett. 2011;107:155704. 22107304 10.1103/PhysRevLett.107.155704

[35] Sengers J, Sengers JL. Thermodynamic behavior of fluids near the critical point. Ann Rev Phys Chem. 1986;37:189–222.

[36] Villamaina D, Trizac E. Thinking outside the box: Fluctuations and finite size effects. Eur J Phys. 2014;35(3): Article 035011.

[37] Divoux T, Fardin MA, Manneville S, Lerouge S. Shear banding of complex fluids. Ann Rev Fluid Mech. 2016;48:81–103.

[38] Han M, Yan J, Granick S, Luijten E. Effective temperature concept evaluated in an active colloid mixture. Proc Natl Acad Sci U S A. 2017;114(29):7513–7518. 28674007 10.1073/pnas.1706702114 PMC5530701

[39] Banerjee D, Vitelli V, Jülicher F, Surówka P. Active viscoelasticity of odd materials. Phys Rev Lett. 2021;126(13):138001. 33861116 10.1103/PhysRevLett.126.138001

[40] Lobmeyer DM, Biswal SL. Grain boundary dynamics driven by magnetically induced circulation at the void interface of 2D colloidal crystals. Sci Adv. 2022;8(22): Article eabn5715. 35658046 10.1126/sciadv.abn5715 PMC9166398

[41] Liu P, Zhu H, Zeng Y, du G, Ning L, Wang D, Chen K, Lu Y, Zheng N, Ye F, et al. Oscillating collective motion of active rotors in confinement. Proc Natl Acad Sci U S A. 2020;117(22):11901–11907. 32430333 10.1073/pnas.1922633117 PMC7275704

[42] Scholz C, Engel M, Pöschel T, Pöschel T. Rotating robots move collectively and self-organize. Nat Commun. 2018;9(1):931. 29500429 10.1038/s41467-018-03154-7 PMC5834624

[43] Yang X, Ren C, Cheng K, Zhang H. Robust boundary flow in chiral active fluid. Phys Rev E. 2020;101(2-1): Article 022603. 32168608 10.1103/PhysRevE.101.022603

[44] Petroff AP, Wu XL, Libchaber A. Fast-moving bacteria self-organize into active two dimensional crystals of rotating cells. Phys Rev Lett. 2015;114(15):158102. 25933342 10.1103/PhysRevLett.114.158102

[45] Chen X, Yang X, Yang M, Zhang HP, Zhang H. Dynamic clustering in suspension of motile bacteria. EPL. 2015;111(5):54002.

[46] Riedel IH, Kruse K, Howard J. A self-organized vortex array of hydrodynamically entrained sperm cells. Science. 2005;309(5732):300–303. 16002619 10.1126/science.1110329

[47] Wang X, Chen S, Nan H, Liu R, Ding Y, Song K, Shuai J, Fan Q, Zheng Y, Ye F, et al. Abnormal aggregation of invasive cancer cells induced by collective polarization and ECM-mediated mechanical coupling in coculture systems. Research. 2021; Article 9893131. 34957406 10.34133/2021/9893131 PMC8678614

[48] Palacci J, Sacanna S, Steinberg AP, Pine DJ, Chaikin PM. Living crystals of light-activated colloidal surfers. Science. 2013;339(6122):936–940. 23371555 10.1126/science.1230020

[49] Che S, Zhang J, Mou F, Guo X, Kauffman JE, Sen A, Guan J. Light-programmable assemblies of isotropic micromotors. Research. 2022;2022: Article 9816562. 35928302 10.34133/2022/9816562 PMC9297725

[50] Li L, Yu Z, Liu J, Yang M, Shi G, Feng Z, Luo W, Ma H, Guan J, Mou F. Swarming responsive photonic nanorobots for motile-targeting microenvironmental mapping and mapping-guided photothermal treatment. NanoMicro Lett. 2023;15(1):141. 37247162 10.1007/s40820-023-01095-5 PMC10226971

[51] Wang L, Gao H, Sun H, Ji Y, Song L, Jia L, Wang C, Li C, Zhang D, Xu Y, et al. Reconfigurable vortex-like paramagnetic nanoparticle swarm with upstream motility and high body-length ratio velocity. Research. 2023;6:0088. 36996337 10.34133/research.0088 PMC10042322

