# Supplementary material for: Odd Response-Induced Phase Separation of Active Spinners
Source: Research (Wash D C). 2024 May 3;7:0356. doi: 10.34133/research.0356 (PMC11075672; doi:10.34133/research.0356)
Supplement: Supplementary 1 — Figs. S1 to S5 Movies S1 to S3 [file research.0356.f1.zip › SI(clean).pdf]

Supplementary material for "Odd response-induced phase separation of active spinners"

Yu Ding,[1,2] Boyi Wang,[1,2] Qing Yang,[1,3] Zhiyuan Zhao,[3,4] Shigeyuki Komura,[3,4,5] Ryohei Seto,[3,4,6] Mingcheng Yang,[1,2,7,*] and Fangfu Ye[1,2,3,4,7,†]

[1]*Beijing National Laboratory for Condensed Matter Physics and Laboratory of Soft Matter Physics, Institute of Physics, Chinese Academy of Sciences, Beijing 100190, China*

[2]*School of Physical Sciences, University of Chinese Academy of Sciences, Beijing 100049, China*

[3]*Wenzhou Institute, University of Chinese Academy of Sciences, Wenzhou, Zhejiang 325001, China*

[4]*Oujiang Laboratory, Wenzhou, Zhejiang 325000, China*

[5]*Department of Chemistry, Graduate School of Science, Tokyo Metropolitan University, Hachioji, Tokyo 192-0397, Japan*

[6]*Graduate School of Information Science, University of Hyogo, Kobe, Hyogo 650-0047, Japan*

[7]*Songshan Lake Materials Laboratory, Dongguan, Guangdong 523808, China*

I. SIMULATION METHOD

The 2D active spinner fluid consists of N spinning disk-like particles of radius a and mass m in a square box, with the periodic boundary condition in both x and y directions. In simulations, the box area remains fixed, $L^2 = (100a)^2$, and the packing fraction $\rho = \pi a^2 N/L^2$ is adjusted by changing the number of the spinners. They interact via the Weeks-Chandler-Andersen type of potential, $U(r) = 4\epsilon[(\frac{2a}{r})^{24} - (\frac{2a}{r})^{12}] + \epsilon$ if $r < 2^{1/12}(2a)$ and 0 otherwise. Besides, neighboring particles also couple through tangential friction realized via the rough disk collision (bounce-back collision) [1], that results in non-conservative transverse interaction. Throughout the paper, lengths are in units of a, masses in units of m, and energies in units of ϵ, corresponding to setting $a = 1$, $m = 1$, and $\epsilon = 1$. Thus, time is expressed in unit of $t_0 = \sqrt{ma^2/\epsilon}$, torque in unit of ϵ, temperature in unit of ϵ/k_{B}, and translational friction coefficient in unit of $\sqrt{m\epsilon/a^2}$.

The translational dynamics of the particles evolves according to the underdamped Langevin equation,

$$m\dot{\mathbf{v}}_i = \mathbf{F}_{\mathtt{p}} + \mathbf{F}_{\mathtt{ex}} + \boldsymbol{\zeta} - \gamma_t \mathbf{v}_i, \tag{1}$$

with $\mathbf{v}_i$ the velocity of the ith particle, $\gamma_t = 100\sqrt{m\epsilon/a^2}$ the translational friction coefficient, and $\mathbf{F}_{\mathtt{p}}$ the interparticle Weeks-Chandler-Andersen-type repulsion. The stochastic force $\boldsymbol{\zeta}$ is Gaussian distributed with $\langle \boldsymbol{\zeta}(t)\rangle = \mathbf{0}$ and $\langle \boldsymbol{\zeta}(t)\boldsymbol{\zeta}(t')\rangle = 2k_{\mathrm{B}}T\gamma_t\delta(t-t')\mathbf{1}$, with the temperature T. In the main text, we explore the phase diagram of the spinner fluid in the temperature range $[0.01, 0.15]$. The rotational dynamics of the particles develops according to

$$I\dot{\omega}_i = \mathrm{T}_{\mathtt{d}} + \xi - \gamma_r \omega_i, \tag{2}$$

where $I = ma^2/2$ is the particle momenta of inertia, ω_i the angular velocity of the ith particle, $\gamma_r = 4\gamma_t a^2/3$ the rotational friction coefficient, $\mathrm{T}_{\mathtt{d}}$ the active torque, and ξ the Gaussian distributed stochastic torque with $\langle \xi(t)\rangle = 0$ and $\langle \xi(t)\xi(t')\rangle = 2k_{\mathrm{B}}T\gamma_r\delta(t-t')$. The equations of motion are integrated with the time step $\Delta t = 2 \times 10^{-3} t_0$.

The rough disk collision (bounce-back collision) [1] that generates the friction between two particles in contact (say i and j, their separation is smaller than the cutoff of the potential

* mcyang@iphy.ac.cn

† fye@iphy.ac.cn

interaction) is realized by instantaneously updating $\mathbf{v}_i$ and ω_i according to $\delta\mathbf{v}_i = \delta\mathbf{p}_i/m$ and $\delta\omega_i = -\frac{1}{2}(\mathbf{r}_i - \mathbf{r}_j) \times \delta\mathbf{p}_i/I$, at each simulation step. Here, $\mathbf{r}_i$ is the the position of the center of the ith particle. The impulse $\delta\mathbf{p}_i$ is determined by conservation laws,

$$\delta\mathbf{p}_i = -m(\tilde{\mathbf{v}}_{ij}^{\parallel} + \frac{\kappa}{1+\kappa}\tilde{\mathbf{v}}_{ij}^{\perp}), \tag{3}$$

with the parameter $\kappa = 4I/m(\mathbf{r}_i - \mathbf{r}_j)^2$, and $\tilde{\mathbf{v}}_{ij}^{\parallel}$ and $\tilde{\mathbf{v}}_{ij}^{\perp}$ the components of the relative velocity at collision point, $(\mathbf{v}_i - \mathbf{v}_j) - \frac{1}{2}(\omega_i + \omega_j) \times (\mathbf{r}_i - \mathbf{r}_j)$, parallel and perpendicular to $\mathbf{r}_i - \mathbf{r}_j$, respectively. To achieve a large friction between the particles, the parallel operation in the bounce-back collision is not implemented, which still conserves the momentum, angular momentum, and energy in this instantaneous collision.

The chiral active fluid is sheared uniformly by combining the Lees-Edwards boundary condition and an external force field $\mathbf{F}_{\mathbf{ex}} = f(y)\hat{x} = \dot{\gamma}\gamma_t(y - L/2)\hat{x}$, which produces a shear rate $\dot{\gamma}t_0 = 10^{-3}$. Note that, since the particles experience friction from the external environment, the Lees-Edwards boundary condition alone cannot create a uniform shear flow, such that a linear y-dependent external force field needs to be applied.

II. NEGLIGIBLE INERTIAL EFFECT

The underdamped Langevin equations above have been often employed to simulate the active spinners [2–5]. The reason is that the operation for producing the inter-spinner transverse coupling (friction) usually involves instantaneous velocity and angular velocity of the spinner, especially for the spinning disks or spheres, such that the underdamped Langevin equation is a more proper framework.

In order to simulate a regime of low Reynolds number, we choose a large damping coefficient and a small mass and moment of inertia. According to the literatures [6–9], the inertial effect can be quantified by dimensionless numbers τ_m/τ_r and τ_I/τ_r, with $\tau_m = m/\gamma_t$ the translational inertial timescale, $\tau_I = I/\gamma_r$ the rotational inertial timescale and $\tau_r = 1/D_r$ the rotational diffusion timescale. When τ_m/τ_r and τ_I/τ_r are much smaller than 1, the inertia is negligible. In our system, $\tau_m/\tau_r = 1 \times 10^{-5}$ and $\tau_I/\tau_r = 4 \times 10^{-6}$, which are well below the range in which the inertia is significant. Moreover, the flow field and hydrodynamic force obtained from the Langevin equation simulations with similar parameters well agree with those calculated through Stokes-type equation without inertia [10], indicating that the

Reynolds number in our system is low enough and the inertia is negligible.

III. HOW TO CHOOSE THE SHEAR RATE AND THE DRIVING TORQUE

In the simulations, we fix the shear rate $\dot{\gamma}$ and torque $\mathrm{T}_{\mathtt{d}}$. The main reason for this is as follows. The equilibrium phase transitions are usually investigated in the $T-\rho$ plane, and $\dot{\gamma}$ and $\mathrm{T}_{\mathtt{d}}$ do not exist in equilibrium systems. In order to straightforwardly and thoroughly compare with the equilibrium gas-liquid phase separation, we therefore fix $\dot{\gamma}$ and $\mathrm{T}_{\mathtt{d}}$ and change T and ρ in the present simulation of spinner fluid.

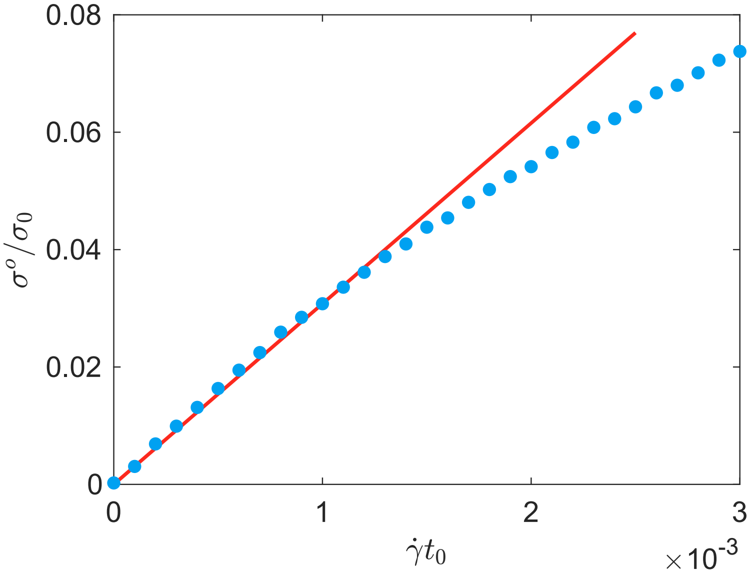

FIG. S1. The relationship between the odd viscosity stress and shear rate. Here, we define $\sigma_0 = m/t_0^2$.

In the simulations, we take the shear rate $\dot{\gamma}t_0 = 10^{-3}$ and the driving torque $\mathrm{T}_{\mathtt{d}}/\epsilon = 40$ (counterclockwise). A proper shear rate must satisfy two conditions: it should be large enough to achieve a good signal-to-noise ratio, while small enough to maintain a linear response of the system. Figure S1 shows that our system remains in the linear regime at $\dot{\gamma}t_0 = 10^{-3}$, having a good signal-to-noise ratio. Moreover, $\dot{\gamma}t_0 = 10^{-3}$ is also consistent with the value taken in the work on spinner fluid by Han et al. [4], where $\dot{\gamma}t_0$ ranges from 8×10^{-4} to 2.5×10^{-3}. For the driving torque, we choose $\mathrm{T}_{\mathtt{d}}/\epsilon = 40$, so that the present spinner fluid has a significant odd viscosity [11] and the resulted angular velocity, $\omega_0 = \mathrm{T}_{\mathtt{d}}t_0/\gamma_r = 0.3$, is still moderate. This value is well within the range of the dimensionless angular velocity,

$\omega_0 \in [0, 2.0]$, in the work by Nguyen et al. [2]. Therefore, both $\dot{\gamma}$ and T_d in our present work compare well to those in the other existing studies.

IV. PHASE DIAGRAM, CRITICAL POINT AND BINODAL CURVE DETERMINED FROM SIMULATIONS

The steady-state non-equilibrium phase diagram [Fig. 1(b) in the main text] is obtained as follows. The simulations are performed from two different types of initial configurations with the same packing fraction: a homogeneous fluid configuration and a dense stripe configuration. During a long simulation period $\sim 10^9\Delta t$, if both initial configurations evolve into a steady phase-separated state, then the system will be classified into the unstable domain; if the uniform fluid configuration survives and the dense stripe develops the phase-separated state, then the corresponding system will be considered metastable; otherwise, if both initial configurations develop a steady-state uniform fluid, the system will be classified into the homogeneous fluid phase. Even for longer simulation time, the likelihood of spontaneous phase separation in metastable fluids remains low, since the spontaneous nucleation requires spanning the entire system in the flow direction (the x-direction) due to the system symmetry such that it hardly happens within the simulation duration.

We determine the location of the critical point in the simulation based on the fluctuation of the particle number in a sub-region of the system, e.g. in sub-region 2 of Fig. S2. As depicted in Fig. S2, the system can be divided into sub-regions separately in the x and y directions, which have different fluctuation behavior.

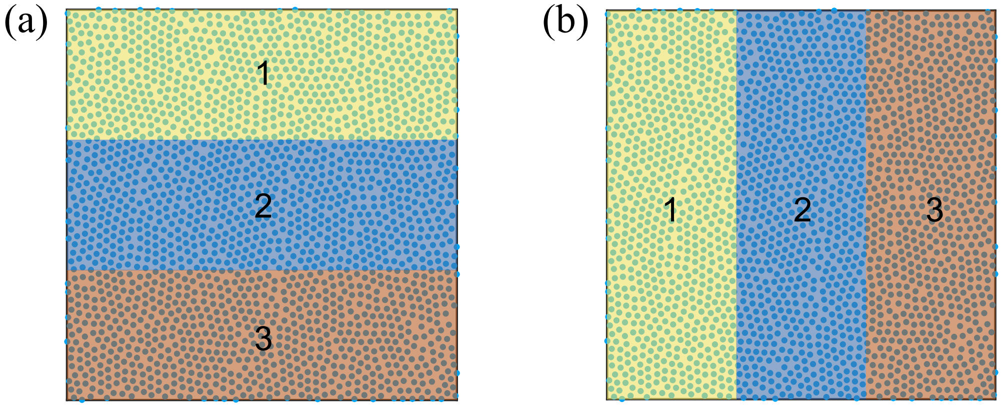

FIG. S2. (a) and (b) represent sub-regions that are subdivided along the y- and x-axes, respectively.

In the simulations, the binodal curve is determined as the envelope of the region, where an initial phase-separated state is stable. Actually, in the metastable region, an initial homogeneous fluid state is also 'stable', because the spontaneous nucleation requires spanning the entire system in the flow direction (the x-direction) due to the system symmetry, such that it hardly happens within the simulation duration. The same method has also been employed to determine the binodal curves for the active Brownian particles [12].

V. ODD VISCOUS STRESS AND PRESSURE OF CHIRAL ACTIVE FLUIDS

The coefficients in Eq. (4) of the main text cannot be directly measured in the unstable (phase-separated) region. In order to obtain these coefficients, we reverse the rotational direction of the spinners (spinning clockwise), while the shear is held constant. The phase separation does not occur in the case of negative active torques (spinning clockwise) [13], where the particles remain uniformly distributed throughout the simulation box [see Fig. S3(a)]. For a negative torque, the odd viscosity η_o^- satisfies the relation $\eta_o^- = -\eta_o$, with η_o the odd viscosity under the positive torque of the same magnitude (employed in the main text) [4, 11, 14]. This allows us to quantify the odd viscosity of spinner fluids in the region of phase separation. We can calculate the stress components by imposing the same shear rate as in the main text. The measured shear flow profile is plotted in Fig. S3(b), showing a constant shear rate throughout the system. As discussed in the main text, the normal stresses across the x and y planes read $\sigma_{xx}^f = -p + \eta_o^- \partial_y v_x$ and $\sigma_{yy}^f = -p - \eta_o^- \partial_y v_x$, respectively. So, the pressure and the odd viscous stress in the y direction for the fluid of spinners rotating counterclockwise are

$$\begin{aligned} p &= -\frac{1}{2}(\sigma_{xx}^f + \sigma_{yy}^f), \\ \sigma_{yy}^{V,o} = -\eta_o \partial_y v_x &= \eta_o^- \partial_y v_x = \frac{1}{2}(\sigma_{xx}^f - \sigma_{yy}^f). \end{aligned} \tag{4}$$

The p and $\sigma_{yy}^{V,o}$ are shown as the blue and yellow lines, respectively, in Fig. 2(a) of the main text. In simulations, the normal stresses σ_{xx}^f and σ_{yy}^f are directly determined by measuring the momentum fluxes across imaginary x and y planes.

We here validate these relationships within a stable uniform fluid environment at a relative high temperature of 0.15 (located at the top of the phase diagram). As demonstrated in Fig. S4, the pressure and odd viscosity stress of the counterclockwise spinners nearly perfectly

overlap with those of the clockwise spinners, so that the measured pressure and viscosity hardly depend on the rotation direction of the spinner.

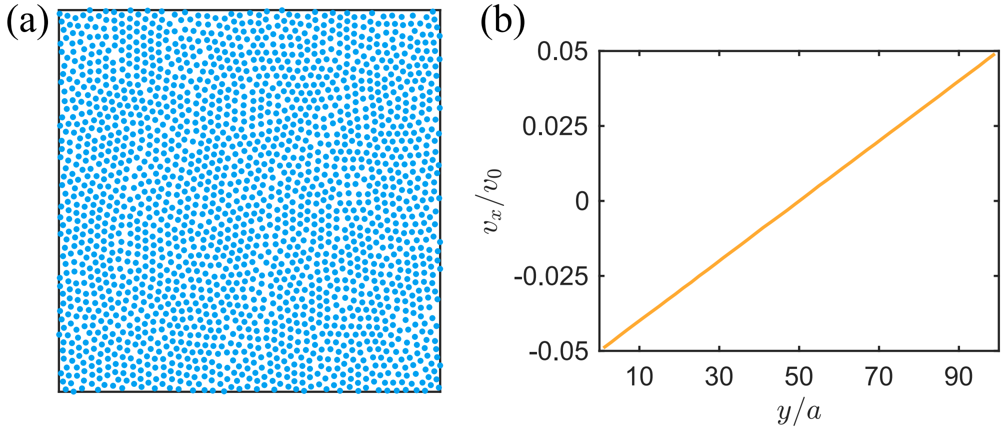

FIG. S3. (a) Active spinner fluid remains a homogeneous fluid phase under a negative active torque. (b) Flow velocity of the spinner fluid, obtained from the simulation, as a function of the y coordinate. Here, we define $v_0 = a/t_0$.

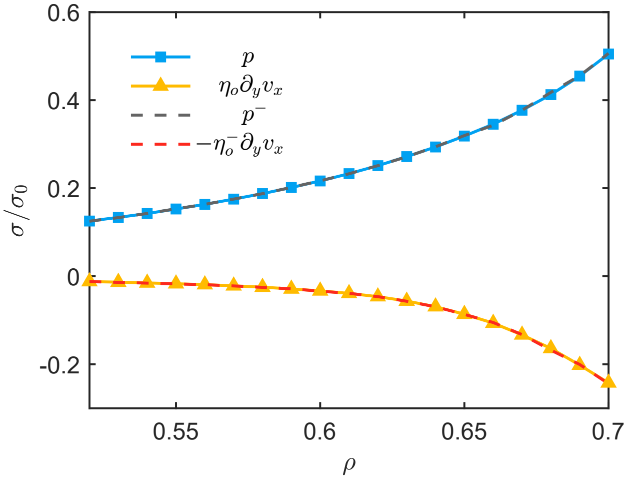

FIG. S4. Normal stress (in unit of $\sigma_0 = m/t_0^2$) vs. ρ, with $T = 0.15$. The solid line and the dashed line correspond to the spinners rotating counterclockwise and clockwise, respectively.

VI. CALCULATION OF THE MAXIMUM AND MINIMUM WIDTHS OF THE SOLID STRIPE

In a shear flow field, the solid stripe, coexisting with the vacuum state, moves as a single unit. In the steady state, the tangential stress inside the solid stripe balances the external body forces on the spinners, i.e., the external driving force $\mathbf{F}_{\texttt{ex}} = f(y)\hat{x} = \dot{\gamma}\gamma_t(y - L/2)\hat{x}$ and the environmental friction (see Fig. S5). Thus, the x-direction force balance on an element of the solid stripe reads $0 = \partial_y \sigma_{xy}(y) + [f(y') - \gamma_t v_0]\rho(y')/V_p$, yielding the tangential stress by integration,

$$\sigma^s_{xy}(y) = \frac{1}{V_p}\int_y^{y_0+w_s} [f(y') - \gamma_t v_0]\rho(y')dy', \tag{5}$$

where $\sigma^s_{xy}(y_0 + w_s) = 0$ is used, since the tangential stress is zero at the outermost edges of the solid stripe due to the vacuum environment. Here, $V_p = \pi a^2$ is the area of a single particle, $\gamma_t v_0$ is single-particle friction from the environment, and y_0 and w_s separately refer to the center and half-width of the stripe. The v_0 is the velocity of the whole stripe, which is determined by $0 = \sigma^s_{xy}(y_0 - w_s) = \frac{1}{V_p}\int_{y_0-w_s}^{y_0+w_s}[f(y') - \gamma_t v_0]\rho(y')dy'$, yielding $v_0 = f(y_0)/\gamma_t$ due to the linear dependence of $f(y)$. Equation (5) well describes the tangential stress distribution inside the stripe and indicates that $\sigma^s_{xy}(y)$ monotonically increases from the stripe edge to the stripe center [see Fig. 3(c) in the main text]. Moreover, in the steady-state stripe that coexists with the vacuum, the normal stress in the y direction (effective pressure) vanishes everywhere, i.e., $p^s_{\texttt{eff}}(\rho(y)) = -\sigma^s_{yy}(\rho(y)) = 0$, which is achieved by spontaneously adjusting the local density. In the following, we combine the elastic theory and independent simulation of uniform spinner crystals (with periodic boundary) to predict the density distribution in the solid stripe.

According to the stress-strain relationship [5, 15], the stress tensor of an uniform spinner solid has the form

$$\begin{aligned}\sigma^s_{ij} &= -p\delta_{ij} + \sigma^{spin}\epsilon_{ij} + \sigma^E_{ij} \\ &= -p\delta_{ij} + 2\eta_R\omega\epsilon_{ij} + \lambda\delta_{ij}\partial_k u_k + \mu(\partial_i u_j + \partial_j u_i - \delta_{ij}\partial_k u_k) \\ &+ K_o(\partial_k\epsilon_{ik}u_j + \partial_i\epsilon_{jk}u_k),\end{aligned} \tag{6}$$

with λ, μ and K_o being the shear, bulk and odd elastic moduli, respectively. Since only $\partial_y u_x$

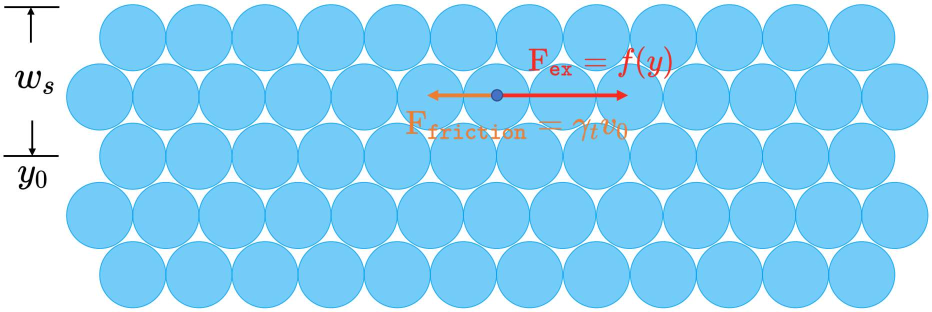

FIG. S5. Schematic of a solid stripe in a simple shear flow field.

is nonzero, then the related stresses are

$$\begin{aligned}\sigma^s_{xy} &= \sigma^{spin} + \sigma^E_{xy} = 2\eta_R\omega + \mu\partial_y u_x,\\ \sigma^s_{yy} &= -p + \sigma^E_{yy} = -p - K_o\partial_y u_x.\end{aligned} \tag{7}$$

By measuring σ^s_{ij} of a periodic hexagonal spinner crystal with a shear strain $\partial_y u_x$ within the elastic limit, we can quantify the elastic modulus of the spinner crystal. From Eq.(7), the normal stress is related to σ^s_{xy} by

$$\sigma^s_{yy}(\rho;\sigma^s_{xy}) = -p - K_o\frac{\sigma^s_{xy} - 2\eta_R\omega}{\mu} = -p^s_{\mathtt{eff}}(\rho;\sigma^s_{xy}). \tag{8}$$

Here, all the elastic moduli and pressure sensitively depend on the system density. Although the properties of the solid stripe are inhomogeneous along the y direction, it is reasonable to consider that Eq.(8) properly describes the local normal stress of the stripe. Therefore, we are able to determine the density distribution of the inhomogeneous stripe, by independently shearing a uniform spinner crystal and finding the solution of $\sigma^s_{yy}(\rho;\sigma^s_{xy}) = 0$, namely $p^s_{\mathtt{eff}}(\rho;\sigma^s_{xy}) = 0$ [see Fig. 3(d) in the main text].

However, the effective pressure cannot be reduced to zero within the elastic limit, when σ^s_{xy} is too low [see the left side of Fig. 3(d) in the main text], since the odd elasticity effect (hence the effective attraction) in this case is too weak to balance the traditional pressure. The minimal tangential stress $\sigma^{s,\mathtt{min}}_{xy}$, that ensures $p^s_{\mathtt{eff}}(\rho;\sigma^s_{xy}) = 0$ is solvable, corresponds to the tangential stress on the secondary outer layer of the narrowest stable stripe (Note that at the outermost edge $\sigma^s_{xy}(y_0 \pm w_s) = 0$.), since $\sigma^s_{xy}(y)$ increases with the stripe width [Eq.(5)]. For smaller w_s (lower σ^s_{xy}), the odd elasticity effect can not stabilize the narrow solid stripe [as displayed by the simulation snapshot in Fig. 4(b) in the main text], and the

stripe loses its stability from its edges. Considering the stripe located in the system's center, $y_0 = L/2$, due to the translational symmetry, from Eq.(5) the minimum width $w_{\mathtt{min}}$ of the solid stripe satisfies the relation,

$$\begin{aligned}\sigma_{xy}^{s,\mathtt{min}} &= \frac{1}{V_p}\int_{\frac{L}{2}+w_{\mathtt{min}}-\sqrt{V_p'}}^{\frac{L}{2}+w_{\mathtt{min}}} 0.8\times f(y')dy' \\ &= \frac{1}{10}\left(\frac{w_{\mathtt{min}}}{\sqrt{V_p'}}-\frac{1}{2}\right),\end{aligned} \tag{9}$$

where $\sqrt{V_p'} = \sqrt{V_p/0.8}$ is the thickness of a monolayer of particles, and $\rho(y)$ is approximated as the stripe average density 0.8. On the other hand, when σ_{xy}^s is very high, the effective pressure cannot decrease to zero within the elastic limit [see the right side of Fig. 3(d) in the main text], otherwise the spinner crystal is broken. This means that a very wide solid stripe is also unstable due to the plastic deformation at its center, as displayed by the simulation snapshot in Fig. 4(c) in the main text. The maximum $\sigma_{xy}^{s,\mathtt{max}}$ in the uniform spinner crystal, which still ensures $p_{\mathtt{eff}}^s(\rho;\sigma_{xy}^s) = 0$ is solvable, corresponds to the tangential stress at the center of the widest stable stripe. Thus, the maximum stripe width $w_{\mathtt{max}}$ is determined as

$$\begin{aligned}\sigma_{xy}^{s,\mathtt{max}} &= \frac{1}{V_p}\int_{\frac{L}{2}}^{\frac{L}{2}+w_{\mathtt{max}}} 0.8\times f(y')dy' \\ &= \frac{1}{20V_p'}w_{\mathtt{max}}^2.\end{aligned} \tag{10}$$

In terms of $\sigma_{xy}^{s,\mathtt{min}}$ and $\sigma_{xy}^{s,\mathtt{max}}$ determined through the uniform spinner crystal, the predicted minimum and maximum stripe widths compare well to the direct simulation measurement in the phase-separated (stripe-vacuum) systems, as shown in Fig. 4(a) in the main text. During direct measurement, we first use a stable stripe as a reference. Then, we determine the maximum width by incrementally adding spinners to the stable stripe until fragmentation is observed within this wider stripe. Similarly, we gradually remove particles layer by layer until the stripe becomes unstable, which determines the minimum width of the stable stripe.

Movie S1 (separate file). Simulation movie at $T = 0.14$, as shown in Figure 1c.

Movie S2 (separate file). Simulation movie at $T = 0.08$, as shown in Figure 1d.

Movie S3 (separate file). Simulation movie at $T = 0.01$, as shown in Figure 1e.

---

[1] M. Allen and D. Tildesley, *Computer Simulation of Liquids* (Clarendon Press, 1989).

[2] N. H. Nguyen, D. Klotsa, M. Engel, and S. C. Glotzer, Physical review letters **112**, 075701 (2014).

[3] P. Liu, H. Zhu, Y. Zeng, G. Du, L. Ning, D. Wang, K. Chen, Y. Lu, N. Zheng, F. Ye, *et al.*, Proceedings of the National Academy of Sciences **117**, 11901 (2020).

[4] M. Han, M. Fruchart, C. Scheibner, S. Vaikuntanathan, J. J. De Pablo, and V. Vitelli, Nature Physics **17**, 1260 (2021).

[5] E. S. Bililign, F. Balboa Usabiaga, Y. A. Ganan, A. Poncet, V. Soni, S. Magkiriadou, M. J. Shelley, D. Bartolo, and W. Irvine, Nature Physics **18**, 212 (2022).

[6] C. Scholz, S. Jahanshahi, A. Ldov, and H. Löwen, Nature communications **9**, 5156 (2018).

[7] S. Mandal, B. Liebchen, and H. Löwen, Physical review letters **123**, 228001 (2019).

[8] H. Löwen, The Journal of chemical physics **152** (2020).

[9] M. Sandoval, Physical Review E **101**, 012606 (2020).

[10] X. Lou, Q. Yang, Y. Ding, P. Liu, K. Chen, X. Zhou, F. Ye, R. Podgornik, and M. Yang, Proceedings of the National Academy of Sciences **119**, e2201279119 (2022).

[11] Q. Yang, H. Zhu, P. Liu, R. Liu, Q. Shi, K. Chen, N. Zheng, F. Ye, and M. Yang, Physical Review Letters **126**, 198001 (2021).

[12] J. Stenhammar, D. Marenduzzo, R. J. Allen, and M. E. Cates, Soft matter **10**, 1489 (2014).

[13] Z. Zhao, B. Wang, S. Komura, M. Yang, F. Ye, and R. Seto, Physical Review Research **3**, 043229 (2021).

[14] T. Markovich and T. C. Lubensky, Physical Review Letters **127**, 048001 (2021).

[15] C. Scheibner, A. Souslov, D. Banerjee, P. Surówka, W. Irvine, and V. Vitelli, Nature Physics **16**, 475 (2020).
